# Retraction: Fructose Mediated Non-Alcoholic Fatty Liver Is Attenuated by HO-1-SIRT1 Module in Murine Hepatocytes and Mice Fed a High Fructose Diet

**DOI:** 10.1371/journal.pone.0259219

**Published:** 2021-11-04

**Authors:** The PLOS ONE Editors

After this article [1] was published, concerns came to light involving data reported in Figs 1, 4, 6, 7 and 8. The concerns include both similarities across figure panels within this article [1], and similarities between blot images in [1] and blot images reported in other articles [2–11]. In many cases, the similar data represent different experimental results in the different figures and/or publications. In light of these issues, *PLOS ONE* has concerns about the integrity and reliability of the results and conclusions reported in [1].

The corresponding author requested retraction of this article. They stated that data underlying some of the article’s results are currently held by Marshall University, and that the original data underlying Figs 4A and 7A are not available.

Due to the extensive image concerns and the author’s comments regarding data availability, *PLOS ONE* concluded that the results and conclusions reported in the article are not reliable. Therefore, the *PLOS ONE* Editors retract this article.

The article’s authors either could not be reached or did not respond directly to comment on the retraction decision.

The following figure panels in [1] appear similar to material in other published articles [2–11]. References 2, 3, and 6–11 were published prior to [1] and by different author groups, although some authors overlap with [1]; four of the prior articles [7, 8, 10, 11] are not offered as Open Access articles under a Creative Commons license. For these reasons, the figure panels in [1] that are similar to content in [2, 3, 6–11] are excluded from the *PLOS ONE* article’s [1] license. At the time of retraction, the *PLOS ONE* article [1] was republished to note these exclusions in the figure legends and copyright statement. Readers should refer to the indicated publications for the copyright notices.

- Actin blot in Fig 1E of [1]: similar to previously published content in [11]
- Actin blots in Fig 1F, 6A, 6F, 8B, 8C of [1]: similar to one another and previously published content in [2– 6, 9, 10]
- Actin blots in Figs 6B and 7C of [1]: similar to one another and previously published content in [3, 5]
- Actin blot in Fig 6D of [1]: similar to previously published content in [3, 7–9]
- Actin blot in Fig 7D: similar to content published in [5]
- Actin blot in Fig 8A of [1]: similar to previously published content in [4, 11]
- Fig 1E SIRT1 and Fig 6E FAS panels in [1]: similar to one another and previously published content in [4, 10]
- AMPK panel in Fig 2A and Actin blot in Fig 6E of [1]: similar to one another and previously published content in [4, 10]
- Top left panel (WT) in Fig 4A of [1]: similar to previously published content in [6]
- G6Pase in Fig 6D of [1]: similar to previously published content in [3, 11]

Note, the article cited in this notice as Ref. 11 was retracted in 2021; see [12] for the retraction notice.
